# 3D grain reconstruction from laboratory diffraction contrast tomography

**DOI:** 10.1107/S1600576719005442

**Published:** 2019-05-31

**Authors:** Florian Bachmann, Hrishikesh Bale, Nicolas Gueninchault, Christian Holzner, Erik Mejdal Lauridsen

**Affiliations:** a Xnovo Technology ApS, Theilgaards Alle 9, 1th., Køoge, 4600, Denmark; b Carl Zeiss X-ray Microscopy, 4385 Hopyard Road, Pleasanton, CA 94588, USA

**Keywords:** three-dimensional X-ray diffraction (3DXRD), grain mapping, DCT, X-ray diffraction contrast microscopy, reconstruction schemes

## Abstract

A method for reconstructing the three-dimensional grain structure from data collected with a recently introduced laboratory-based X-ray diffraction contrast tomography system is presented. Diffraction contrast patterns are recorded in Laue-focusing geometry. The diffraction geometry exposes shape information within recorded diffraction spots. In order to yield the three-dimensional crystallographic microstructure, diffraction spots are extracted and fed into a reconstruction scheme. The scheme successively traverses and refines solution space until a reasonable reconstruction is reached. This unique reconstruction approach produces results efficiently and fast for well suited samples.

## Abstract 2

A novel reconstruction method to retrieve grain structure from laboratory diffraction contrast tomography is presented and evaluated.

## 1. Introduction   

Over the past two decades, nondestructive volumetric orientation imaging techniques based on X-ray diffraction microscopy have evolved into well established tools for microstructure characterization of polycrystalline materials. Techniques originating at synchrotrons, able to spatially resolve phase, crystallographic orientation, stress and strain in the sample, have been demonstrated to be valuable for studying spatio-temporal relationships between crystallographic microstructure and material behaviour (Schmidt *et al.*, 2004 ▸; King *et al.*, 2008 ▸; Herbig *et al.*, 2011 ▸). Utilizing a monochromatic high-energy parallel synchrotron beam, three-dimensional X-ray diffraction (3DXRD) (Poulsen *et al.*, 1997 ▸, 2001 ▸) and its variants high-energy diffraction microscopy (Suter *et al.*, 2006 ▸; Bernier *et al.*, 2011 ▸; Li & Suter, 2013 ▸), scanning 3DXRD (Hayashi *et al.*, 2015 ▸) and in particular X-ray diffraction contrast tomography (DCT) (Johnson *et al.*, 2008 ▸; Ludwig, Reischig *et al.*, 2009 ▸) have been shown to produce grain maps resolving grain-averaged orientation, shape and strain down to a minimum grain size of a few tens of micrometres. Lately, attempts to access intra-granular orientation (Li *et al.*, 2012 ▸), also referred to as the full orientation field (Viganò *et al.*, 2014 ▸, 2016 ▸), have been made. At the expense of probing volume, it has been shown that differential aperture X-ray microscopy (Larson *et al.*, 2002 ▸; Ice *et al.*, 2005 ▸) can resolve the orientation and strain field down to sub-micrometre level utilizing a polychromatic focused X-ray beam. Other approaches for grain mapping using a synchrotron source have been reported (Bleuet *et al.*, 2008 ▸; Hofmann, Abbey *et al.*, 2012 ▸; Hofmann, Song *et al.*, 2012 ▸; Sanchez *et al.*, 2014 ▸; Ferreira Sanchez *et al.*, 2015 ▸).

The DCT technique (Ludwig *et al.*, 2008 ▸; Johnson *et al.*, 2008 ▸) has recently been adapted to laboratory scale. This adaptation, known as laboratory diffraction contrast tomography (LabDCT) (King *et al.*, 2013 ▸; van Aarle *et al.*, 2015 ▸), has been made commercially available as an additional imaging modality on an X-ray microscope (Holzner *et al.*, 2016 ▸; McDonald *et al.*, 2015 ▸). A polychromatic divergent cone beam emitted by a laboratory micro-focus X-ray source illuminates a millimetre-sized sample. Diffracting grains in the sample form astigmatically magnified spots on the detector (King *et al.*, 2013 ▸). Whereas King *et al.* (2013 ▸) used a rather large magnification, where the source-to-rotation-axis and rotation-axis-to-detector ratio ranges were of the order of 1:5–1:25, McDonald *et al.* (2015 ▸) utilized the Laue-focusing geometry with a 1:1 ratio, resulting in diffraction patterns with elongated diffraction spots. Once grain centroid positions and crystallographic orientations have been found, the shapes of grains are typically reconstructed with algebraic reconstruction techniques (Poulsen & Fu, 2003 ▸; Ludwig, King *et al.*, 2009 ▸; King *et al.*, 2010 ▸; Viganò *et al.*, 2014 ▸) like the simultaneous iterative reconstruction technique (King *et al.*, 2013 ▸; van Aarle *et al.*, 2015 ▸), formulating a system of equations with a suitable forward projector and minimizing an objective function in some sense. Another approach involves maximizing a confidence function (Li & Suter, 2013 ▸), also known as a completeness function, which is the ratio between the observed and expected number of reciprocal vectors associated with a grain orientation (Poulsen *et al.*, 2001 ▸; Schmidt, 2014 ▸).

Here, a versatile reconstruction scheme for grain mapping, named fast geometric indexing, is presented. A simplified diffraction model of a polycrystalline microstructure is considered, wherein acquired data are reduced to binarized diffraction contrast patterns. Spot intensities as well as interactions between grains are neglected and scattering contributions of individual grains are treated independently. The diffraction geometry becomes essential and is rigorously exploited in order to optimize the crystallographic orientation for each grain occupying space by maximizing its completeness. As a consequence, the reconstruction scheme systematically traverses sample space, successively indexing and mapping out grain by grain. Finally, the implementation of the reconstruction scheme is tailored and demonstrated specifically for LabDCT data. The reconstruction scheme extends the previously reported capabilities of extracting grain-based centroid and crystallographic orientation information from LabDCT data recorded in Laue-focusing geometry (McDonald *et al.*, 2015 ▸); now, the morphology of the grains can also be resolved, rendering a full 3D crystallographic microstructure.

## 2. Laboratory diffraction contrast tomography   

### 2.1. X-ray diffraction imaging   

Contrary to conventional absorption contrast tomography, which deals with geodesic beam paths and gives rise to the Radon transform, diffraction contrast tomography addresses diffracted beam paths, where the signal is a superposition of the rather complex diffraction patterns of a crystalline sample.

**Figure 1:**
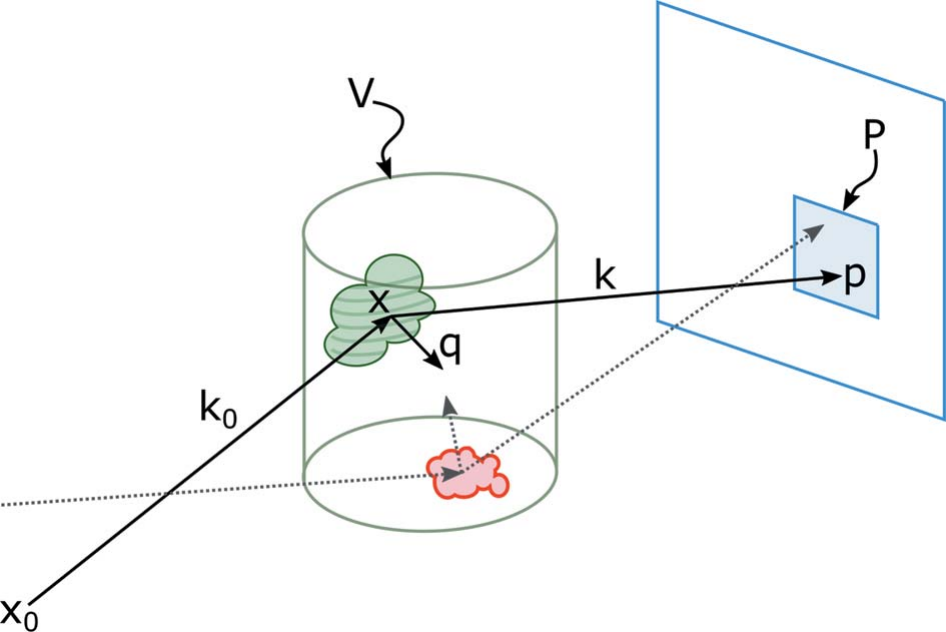
Sketch of diffraction geometry. Pixel *P* counts the integral intensity of all grains in reflection condition contributing from all over the volume *V*.

The signal response of a detection surface area *P*, *i.e.* a detector pixel, is related to the integral intensity
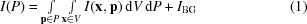
of the diffracted intensity contributions 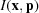 from location 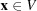 of an illuminated volume *V* of a sample pointing to 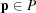, as illustrated in Fig. 1 ▸, and a non-diffracting background intensity *I*_BG_. Consider an incident wavevector 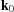 and a reflected one 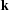, originating from a source point 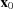, being diffracted at location 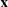 and being detected at 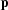, *i.e.*

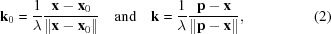
with wavelength λ or X-ray energy *E* = *hc*/λ. Diffraction occurs only if the scattering vector
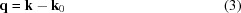
coincides with a reciprocal-space vector 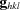 of the crystal lattice at location 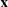 satisfying the necessary Laue diffraction condition
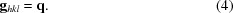
With Bragg’s law this holds for a wavelength
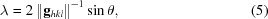
where
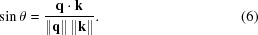
The reciprocal-space vector
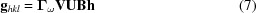
arises from a crystal lattice plane 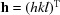 oriented in space accordingly. The reciprocal-space vector 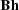 of a single crystal with reciprocal basis matrix 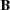 is first rotated by a crystallographic orientation 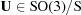, and then possibly a stretch tensor 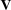 and finally a rigid-body rotation 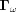 of the sample are applied. The order of multiplication of the rotation and stretch tensors is exchangeable (Bernier *et al.*, 2011 ▸) and depends on the point of view. Computation of 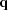 given 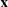 and 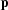 is usually referred to as back projection, whereas computation of 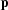 given 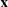 and 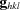 exploiting the reflection
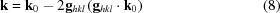
is called forward projection (van Aarle *et al.*, 2015 ▸). Notably, 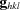 and 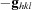 result in the same reflection.

Let the indicator function 1_**g**_*hkl*__(**q**) denote the condition that a crystal lattice plane (*hkl*) diffracts:
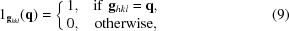
where 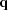 is the back projection with regard to 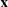 and 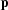 computed from (3). Then, a simplified model for diffracted intensities 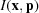 from (1) can be formalized as the integral intensity,
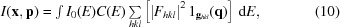
of a polychromatic beam with an X-ray energy intensity distribution *I*_0_(*E*). The sum goes over all crystal lattice planes 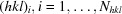, satisfying the diffraction condition (4), and |*F*_*hkl*_|^2^ is the scattering amplitude of the structure factor *F*_*hkl*_. The intensity correction factor *C*(*E*) = *A*(*E*)*G*(*E*)*D*(*E*) may include an energy-dependent attenuation intensity correction *A*(*E*) along the diffracted beam path of the penetrated volume, polarization or geometric intensity corrections *G*(*E*), and a detector intensity correction *D*(*E*) related to the detection sensitivity and efficiency of the used hardware or other effects like point-spread response. Because of the rather selective nature of diffraction condition (4), integral (10) can be simplified to
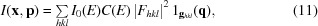
where
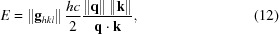
with the entities described above.

### 2.2. Grain mapping   

Traditionally, X-ray diffraction has primarily helped infer the lattice properties of a sample under investigation. With the aid of three-dimensional X-ray diffraction microscopy (Poulsen, 2004 ▸), it is possible to infer a spatially resolved model of the underlying crystallographic microstructure that describes phase, crystallographic orientation, stress and strain or further lattice properties of higher order (Poulsen, 2012 ▸). For grain mapping, the interest is to resolve the grain morphology and the crystallographic orientation in particular. The acquisition strategy seeks to record individual diffraction spots, which can be traced back to the grain of origin.

Several suggestions for experimental setups exist (Lauridsen *et al.*, 2001 ▸; Larson *et al.*, 2002 ▸; Suter *et al.*, 2006 ▸; Johnson *et al.*, 2008 ▸; Ludwig *et al.*, 2008 ▸; King *et al.*, 2013 ▸; Ferreira Sanchez *et al.*, 2015 ▸; McDonald *et al.*, 2015 ▸), which basically differ in the manner of beam formation in order to achieve individual diffraction spots and simplify the problem (1) with its model (10) further. Basic choices affecting the illuminated volume and diffraction geometry include a parallel pencil, line or box beam or a divergent fan or cone beam. The choice of X-ray energy spectrum controls the range of reciprocal-space vectors to satisfy the Laue diffraction condition in the polychromatic case or the Bragg diffraction condition in the monochromatic case. Also, it matters whether the detector is positioned in back-reflection or transmission mode. The experimental setup is targeted to emphasize specific properties pertaining to the investigated crystalline microstructure.

Regardless of which constraints are imposed on the experimental setup in order to acquire data of the type described by integral (1), its reconstruction is notoriously ill-posed. The problems are the imposed Laue class due to Friedel’s law (Friedel, 1913 ▸), the indistinguishable superposition of diffraction signal (Sørensen *et al.*, 2012 ▸), and the loss of signal caused by erroneous or incomplete sampling. The experimental setup greatly influences the design and implementation of reconstruction algorithms to resolve the underlying crystallographic microstructure.

Quantifying the intensity of the diffracted signal is a complex step when considering a polychromatic X-ray beam. Whereas for monochromatic X-rays with energy *E* the source intensity *I*_0_(*E*) and detection intensity correction *D*(*E*) presumably remain constant, in the case of polychromatic X-rays these intensity terms could be difficult to determine experimentally and might only be known insufficiently. Moreover, incorporating absorption along the beam path might become cumbersome, since the X-ray energies in (12) presumably have to be deduced from the diffraction condition, involving assumptions about 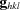. Furthermore, a significant amount of background intensity *I*_BG_ might arise from sample scattering or fluorescence. Hence, it is appealing to replace problem (1) defining the intensity of a pixel *I*(*P*) by the binary problem
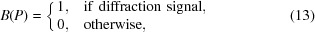
or
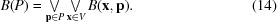
Here the diffracted intensities 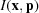 are replaced by
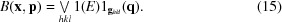
1(*E*) is an indicator function for a valid X-ray energy range, with 1(*E*) = 1 if *E*_min_ < *E* < *E*_max_ and zero otherwise. Binarization immediately implies that data should be acquired such that indistinguishable contributions of several grains, *i.e.* diffraction spot overlap, are mostly omitted.

Given a series of observed binarized diffraction images 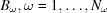, obtained from an experiment, the completeness ratio
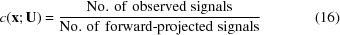
at location 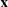 given crystallographic orientation 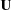 quantifies how much signal can be explained by 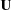. More formally,
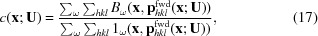
where the forward projection 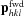 at location **x** for a set of reciprocal space vectors 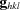 of crystal lattice planes 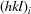, 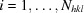, determined by 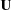 intersects the observed binarized diffraction image *B*_ω_ and the virtual image 1_ω_. Then, the crystallographic orientation 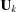 is the optimal solution set of the optimization problem
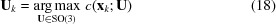
at location 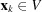, generally referred to as indexing. Maximizing (18) in the monochromatic case has been addressed elsewhere (Lauridsen *et al.*, 2001 ▸; Ludwig, Reischig *et al.*, 2009 ▸; Moscicki *et al.*, 2009 ▸; Bernier *et al.*, 2011 ▸; Sharma *et al.*, 2012 ▸; Li & Suter, 2013 ▸; Schmidt, 2014 ▸). The general case with polychromatic X-rays adds degrees of freedom between the spatial location in the sample and the corresponding diffraction spots as they pass through an X-ray energy range. Nevertheless, a back-projection approach is still applicable for both a divergent and a parallel beam, from which high-confidence lattice plane normals can be employed to index crystallographic orientations using a local optimization scheme.

Assuming a perfect crystal lattice over a grain, *i.e.* a constant crystallographic orientation, let
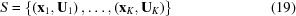
be a seed list of centroid positions 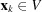 and the associated crystallographic orientations 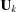 of grains 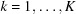 contained in the volume *V*. Then, the crystallographic microstructure to reconstruct can be represented as a partition
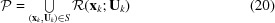
into regions
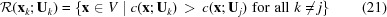
of highest completeness. The reconstruction problem is then to identify centroid positions and their associated crystallographic orientations.

### 2.3. Fast geometric indexing   

Notably, neglecting intensity results in a purely geometric approach without the need for exhaustive intensity calculations concerning the diffracting volume. Moreover, individual contributions of the diffracting volume are treated independently of each other. In order to reconstruct (20), the illuminated volume can be traversed successively over candidate seeds, identifying grains one by one.

Since the seed list *S* of centroid positions and crystallographic orientations is *a priori* unknown, an appropriate algorithm design accounts for systematic sampling of locations in the illuminated volume, achieved by a subdivision scheme. The heuristic approximation algorithm in the following is based on the strategy to successively choose seed locations suggested by the subdivision scheme and to identify adjacent grains and fill the space occupied by them using the input parameters minimum completeness *c*_min_, trust completeness *c*_trust_ and drop-off δ. In order to avoid multiple consecutive draws of the same grain filling the space, the trust completeness *c*_trust_ is intended to circumvent locations where a correct solution can be taken for granted. In contrast, the minimum completeness *c*_min_ is intended to reject solutions if the crystallographic orientation of the maximized completeness is uncertain and too ambiguous. The drop-off parameter δ sets a lower prediction bound to the completeness controlling the presumed size of the region occupied by the anticipated grains of the partition 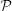. The reconstruction scheme goes as follows:

*Algorithm (fast geometric indexing)*

(0) Put 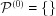, 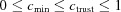, 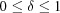, 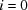. Choose a set of locations 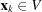, 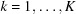 from an arbitrary grid.

(1) Select an unvisited location 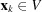 in volume *V* with the lowest completeness 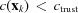.

(2) Optimize
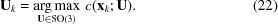
If completeness 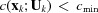 then go to (1).

(3) Grow region
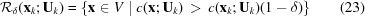
with a drop-off δ around 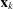.

(4) Compute the weighted centre of mass
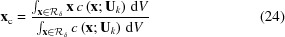
of the grown region 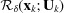.

(5) If 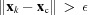 for a distance tolerance 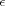 then go to (2) with replacement 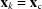, else update partition 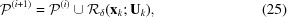
increment 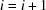 and go to (1).

Depending on the selected location 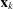, for instance if 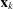 was randomly chosen close to a grain boundary, optimizing 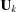 in step (2) might result in an ambiguous solution set of possible candidates of crystallographic orientations. Growing a region around the *n* topmost distinct candidate solutions in step (3) might actually deliver several regions at the same iteration step. Computation of the weighted centre of mass in step (4) moves the initial location iteratively to the optimal one. Note that the resulting partition 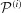 of this heuristic approach depends to some degree on the order of traversal.

The resulting partition 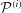 tends to be over partitioned, particularly in regions that are difficult to index. In such regions, optimization of 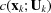 is repeated multiple times because the trust completeness *c*_trust_ cannot be overcome owing to missing information. Merging adjacent regions based on the misorientation angle of their crystallographic orientation (Bachmann *et al.*, 2011 ▸) is a recommended post-processing procedure to identify the actual grains. Because of the model-based assumption of a constant crystallographic orientation within a region, this step should also be accompanied by a re-computation of the weighted mean orientation (Bachmann *et al.*, 2010 ▸). Consequently, the completeness 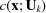 within a newly obtained merged region must also be updated in order to correspond to the diffraction spots within the binarized diffraction contrast patterns.

### 2.4. Data acquisition   

The grain-mapping technique has been implemented in the laboratory on a ZEISS Xradia 520 Versa X-ray microscope (McDonald *et al.*, 2015 ▸). The X-ray microscope is equipped with an additional specialized LabDCT imaging modality optimized to record diffraction contrast patterns. Data acquisition is usually performed in two steps. First, conventional absorption contrast tomography is performed in order to determine the illuminated sample volume *V*. Subsequently, diffraction contrast tomography with the identical acquisition geometry is performed with regard to the same illuminated sample volume *V* but recording diffraction contrast patterns.

**Figure 2:**
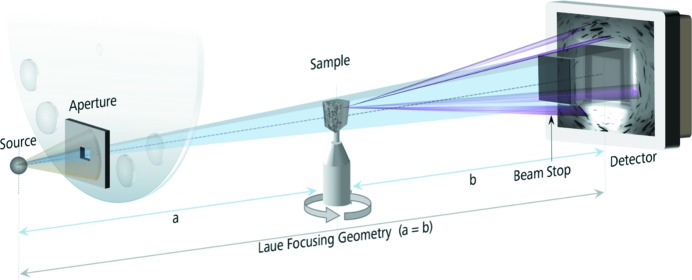
Setup of the LabDCT imaging modality.

The X-ray source of the instrument is a transmission-type micro-focus tube with a tungsten anode, producing a polychromatic white cone beam. Though present, the characteristic emission lines are negligible features of the *Bremsstrahlung* spectrum, ranging from 10 to 160 keV. The sample is placed on a micro-positioning rotation stage between the source and the detector. A scintillator optically coupled to a high-sensitivity CCD camera with an effective pixel size of roughly 3.4 µm detects X-rays transmitted, scattered and diffracted by the sample. The setup of the LabDCT imaging modality is sketched in Fig. 2 ▸. In order to enhance the contrast of the diffracted signal, an aperture mounted in front of the X-ray source defines a cone beam such that for the most part only the sample is illuminated. A centred beam stop shields the high-sensitivity scintillator from overexposure by the direct X-ray beam, and the diffracted signal is recorded in the remaining part. Usually 181 or more diffraction contrast patterns are acquired by rotating the sample once around in 2° or finer steps. Typical working distances range from 12 to 25 mm equidistant from source to rotation axis and rotation axis to detector. In terms of absorption contrast tomography, this symmetric 1:1 distance ratio corresponds to a geometric magnification of factor 2. Uniquely associated with the divergent polychromatic cone beam, aligned lattice domains in the sample that form a grain and that satisfy the Laue diffraction condition act like lenses that bundle and converge the diffracted X-ray beam to a focal zone (Guinier & Tennevin, 1949 ▸) in which the detector is placed. Typical diffraction contrast patterns recorded with this Laue-focusing geometry form elongated diffraction spots on the detector. The advantage of exploiting this Laue-focusing effect is that a higher number of Laue spots can be recorded with less overlap and a higher signal-to-noise ratio on the same projection. This is because a spot covers less space with higher integral intensity, and consequently a larger volume with shorter exposure times can be illuminated.

To accommodate imaging of a variety of different samples, a set of different-sized apertures and beam stops provides further means to adjust the illuminated volume and pattern quality in order to control the information content of diffraction contrast patterns formed on the detector.

### 2.5. Implementation   

Acquired experimental data need to be prepared for the reconstruction algorithm. Extraction of the scattering volume from the reconstructed absorption contrast volume can be performed with standard segmentation methods. The extraction of binarized diffraction contrast patterns from raw projection images consists of several steps. These include a background correction, by subtracting a background estimated from a running median through the image stack (Johnson *et al.*, 2008 ▸), exclusion of the beam stop area and finally a binarization of the pre-corrected diffraction contrast patterns, *e.g.* using a Laplacian of Gaussian segmentation approach (Lind, 2013 ▸). The latter is a crucial step and requires the operator’s best judgement, such that the binarization does not compromise the shape profile of the spots, as it ultimately influences the completeness and thus the quality of the reconstruction.

The crystallography of the sample should be known in advance in order to specify the reciprocal-space vectors required for indexing. In particular, for non-cubic systems, unit-cell lengths and angles affect indexing significantly. As a rule of thumb, more than 15 reciprocal-space vectors are required for the setup described above to yield a reliable grain map that would also allow any possible pseudo-twin orientations to be identified (Schmidt, 2014 ▸). These vectors correspond to the three (body-centred cubic) or four (face-centred cubic, hexagonal close packed) strongest {*hkl*} lattice plane families in the case of cubic or hexagonal symmetry.

The fast geometric indexing reconstruction algorithm described above is discretized on a voxelated volume of arbitrary choice. The set of locations to be traversed systematically is realized by means of a top-down hierarchical cubic close-packing subdivision scheme put onto the voxelated volume. The subdivision results in a set of levels, where the set of locations for each level *L* is such that the Voronoi cell volume associated with the sampling locations is proportional to 2 × 8^*L*^ voxels. Locations of the top level are traversed first, and the reconstruction is successively refined level by level. The procedures are implemented in the workflow-based commercially available software package *GrainMapper3D* (https://xnovotech.com/3d-crystallographic-imaging-software/).

## 3. Results   

**Figure 3:**
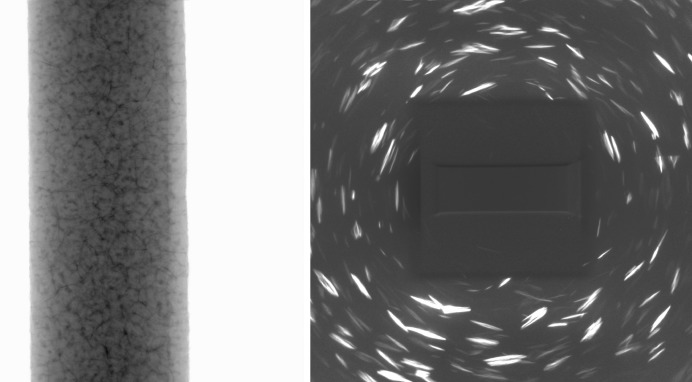
(Left) Acquired absorption projection and (right) diffraction contrast pattern.

Collection of data was performed with a ZEISS Xradia 520 Versa X-ray microscope equipped with a LabDCT imaging module. An aluminium sample with grain boundaries decorated with copper (*cf*. Dake *et al.*, 2016 ▸) was chosen as a model material system to demonstrate the capabilities of LabDCT using the algorithm described. The sample was shaped into the form of a cylindrical rod with a 1400 µm gauge diameter. The cylindrical sample was mounted on a standard pin vise, placed on the sample rotation stage, and positioned 14 mm away from both source and detector. An absorption scan was acquired with 1601 projections of 1 s exposure time each, within 1.5 h. A LabDCT scan was acquired with 181 projections collected in 2° steps with 300 s exposure time, lasting 15 h 40 min. Both scans were performed at 160 kV accelerating voltage and 62 µA current. For the LabDCT scan, a 250 × 750 µm-sized aperture was placed approximately 8.75 mm in front of the polychromatic point source, illuminating a height of approximately 380 µm and a width of 1100 µm on the axis of rotation of the sample. The DCT scan volume consisted of an illuminated volume of 0.573 mm of the aluminium rod. Parts of the sample located outside of the aperture field of view were effectively illuminated during a 360° rotation in at least 65% of all DCT projections acquired. A 2.5 × 2.5 mm beam stop was used to block the direct beam, as shown in the schematic of Fig. 2 ▸. Example projections taken in both the absorption and the diffraction contrast mode are displayed in Fig. 3 ▸. The effective aperture footprint on the detector is 0.8 × 2.4 mm and the footprint of the beam stop approximately 3.0 × 3.0 mm.

The LabDCT reconstructions were performed with the Xnovo *GrainMapper3D* software package on a workstation equipped with a dual Intel Xeon E5-2650 v3 processor (40 threads) and 128 GB RAM. Diffraction contrast patterns were binarized with an adapted Laplacian of Gaussian spot-extraction method (Lind, 2013 ▸). About 76 500 spots were extracted from the 181 projections, *i.e.* approximately 420 per projection on average. A unit-cell length of 4.0496 Å was used as input for the face-centred cubic aluminium (Wycko, 1963 ▸). The four strongest lattice planes, {111}, {200}, {220} and {311}, were selected for indexing with 4, 3, 6 and 12 reciprocal-space vectors, respectively (half of their multiplicity), summing to 25 in total. Illumination calculations were performed in order to correct the completeness in a partially illuminated sample volume. The initial reconstruction was based on the calibration carried over from the instrument, and then the calibration was improved via self-consistent fitting, minimizing the residuals between observed and forward simulated diffraction spots.

**Figure 4:**
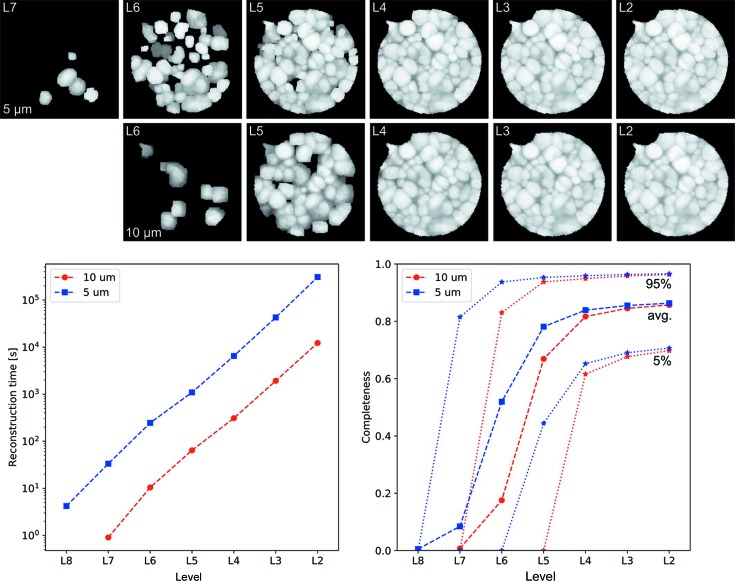
(Top) Completeness maps with 5 and 10 µm resolution through reconstruction levels L7 to L2 and their corresponding (bottom left) reconstruction time and (bottom right) average completeness, 5 and 95% completeness percentiles.

The reconstruction scheme was tested for two scenarios, where the acquired data volume *V* was subdivided into 160 × 160 × 37 (947 200) and 320 × 320 × 73 (677 3760) voxels with voxel edge lengths of 10 and 5 µm, respectively. Fig. 4 ▸ summarizes the reconstruction performance of each level *L* using the reconstruction parameters trust completeness *c*_trust_ = 85%, minimum completeness *c*_min_ = 45% and drop-off δ = 2%. The reconstruction algorithm starts with coarse sampling locations selected from a top level, such as L7, and proceeds by successively choosing refined sampling locations of the grid up to L2. For the aluminium–copper sample considered here, level L2 of the hierarchical subdivision scheme was completed after 3.5 h and 3 d 14 h for the 10 and 5 µm reconstructions, respectively. Roughly 40 000 and 280 000 locations were selected and indexed in total. On average ∼3.4 iterations per second of the reconstruction algorithm could be performed for 10 µm voxel edge length and ∼0.9 iterations per second for 5 µm voxel edge length, which is a factor of ∼3.5 slower compared to roughly 8× more voxels. The completeness maps in Fig. 4 ▸ show a gradual filling up of the volume, with reconstructed grains going hierarchically down from level to level as reconstruction progresses. After 2–2.5% of the total indexing attempts performed, *i.e.* approximately 1000 indexing attempts, an orientation and its corresponding completeness were assigned to more than 99.5% of all voxels in the reconstruction volume, which was the case after completing L4 for both reconstructions after 5 min 12 s and 1 h 50 min, respectively. Less than 5% of all voxels have a completeness less than 60% after reaching L4, and more than half have a completeness higher than 80%. Most information on the reconstructed crystallographic microstructure is gained after completing indexing of reconstruction level L4. The gain of information from reconstruction level L2 is negligible in proportion to the increase in computational time compared with level L3, completed after 32 min and 11 h 50 min for the 10 and 5 µm reconstructions, respectively. Only a handful of additional small grains were identified at the expense of a 6–7 times longer computation time.

**Figure 5:**
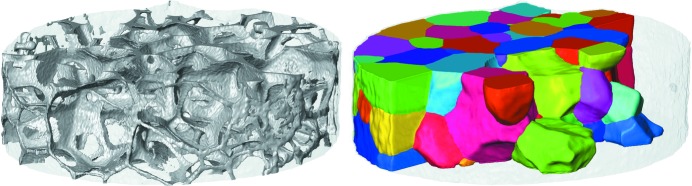
(Left) Absorption reconstruction and (right) DCT reconstruction of the AlCu sample in random colour coding. The illuminated cylindrical volume has a diameter of approximately 1400 µm and a height of 400 µm (volume 0.573 mm).

**Figure 6:**
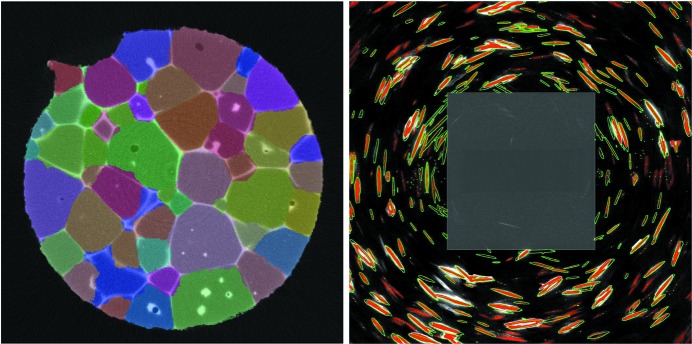
(Left) Overlay of absorption and colour-coded LabDCT reconstruction and (right) binarized diffraction images (red) and forward-projection outline (green) of the diffraction spot.

**Figure 7:**
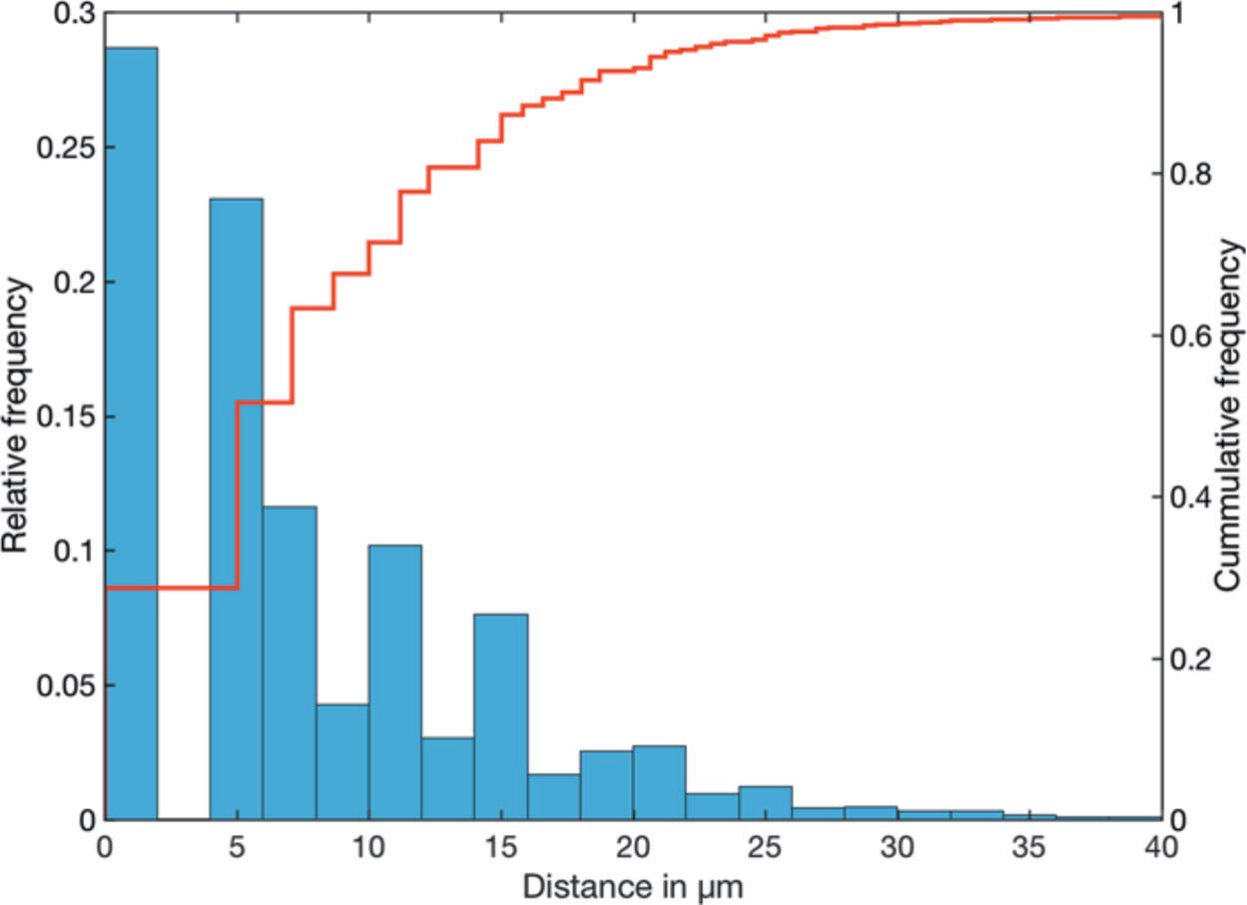
Histogram of the deviation in the grain boundary locations between the absorption and DCT reconstruction.

Fig. 5 ▸ illustrates the copper network along grain boundaries extracted from the reconstruction of absorption contrast data and the polycrystalline microstructure reconstructed from LabDCT data of 129 reconstructed grains from the aluminium–copper sample. The equivalent spherical diameter of the grains is 168 µm on average, including partially illuminated grains at the top and bottom of the sample. The high contrast of the copper phase in the absorption contrast data was used to validate the grain-boundary location assessed from the LabDCT reconstruction. The overlay of the absorption contrast data on top of the LabDCT reconstruction in Fig. 6 ▸ shows a good agreement along grain boundaries. The accuracy of grain boundary location of the LabDCT reconstruction versus the absorption reconstruction is quantified in Fig. 7 ▸, based on the method proposed by Ludwig, Reischig *et al.* (2009 ▸). The average distance in the grain-boundary location between the two reconstructions was found be 7.6 µm (1.52 voxels), whereas 90% fall within a distance of 20 µm (4 voxels).

The forward-projected outline of the first four lattice planes used for indexing of grain boundaries of the reconstructed polycrystalline microstructure in Fig. 6 ▸ explains most of the binarized diffraction spots and reveals a good agreement in position, size and shape. For the whole LabDCT data set, between 155 and 240 (90%) binarized diffractions spots, and 198 on average, were attributed to individual grains. Some of the weaker diffraction spots close to high-intensity spots are not properly binarized, as the adapted Laplacian of Gaussian spot-extraction method did not separate them into individual spots. Unexplained diffraction spots arise either from higher lattice planes or potentially from small grains missed by the indexing, because the signal for higher-order lattice planes is incomplete as a result of their smaller scattering volume.

**Figure 8:**
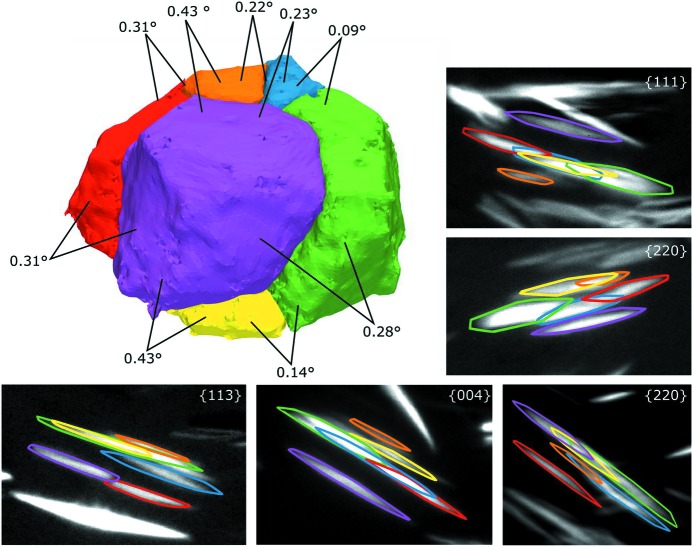
Grain cluster with low-angle grain boundaries and samples of their forward-projected convex hulls, coloured according to the grain.

Some of the high-angle grain boundaries are observed to be only decorated partially with copper, whereas low-angle boundaries are observed generally to be not decorated and could only be revealed by LabDCT. Grain clusters with low-angle boundaries above approximately 0.05° can be distinguished and are validated in the forward projection. An example of such is provided in Fig. 8 ▸, where combinations of diffractions spots of individual grains contributing to a cluster are clearly separable and distinguishable in various projections for different lattice planes, although they partially overlap.

## 4. Discussion   

The presented reconstruction scheme is formulated in a general framework and is independent of the acquisition geometry and X-ray spectrum used. Though only the reconstruction for the LabDCT case with a polychromatic cone beam has been addressed, the reconstruction scheme could be adapted to classical synchrotron 3DXRD or DCT techniques, as these can be seen as special cases utilizing a parallel monochromatic beam. For a low-resolution spatial discretization, the reconstruction scheme is able to produce a meaningful result within minutes, which allows one to fine-tune binarization and reconstruction parameters interactively and on the fly.

Compared with classical synchrotron techniques, similar known limitations on and requirements for samples apply in principle (King *et al.*, 2013 ▸). Grain size, mosaicity or lattice deformation might pose greater limitations, as the polychromatic cone beam forms more complex diffraction contrast patterns and reveals more information captured simultaneously than a monochromatic beam. Owing to the lack of brilliance of a laboratory source compared with a synchrotron source, the minimum scattering volume of grains that is required to form diffraction spots with a suitably high signal-to-noise ratio on the detector is assumed to be at least two or three times the spherical equivalent diameter of what the DCT technique at a synchrotron can resolve. Depending on the diffracted intensities of the material this corresponds to a minimum grain size of the order of >20–40 µm with the instrumentation currently used. The elongation of the diffraction spots on the detector due to the Laue-focusing effect allows for up to 400–500 grains to be illuminated simultaneously while maintaining an acceptable degree of spot overlap (*cf*. McDonald *et al.*, 2015 ▸). Although the current reconstruction model does not account for lattice imperfections across grains, they can be tolerated at the expense of the reliability and quality of the reconstructed grain map up to a certain point, where the diffraction spots formed on the detector are clearly extractable and separable. Lattice defects, deformations and mosaicity immediately affect pattern quality, but can be overcome by reducing the information content by the reducing illuminated volume. Exploiting the Laue-focusing geometry increases the angular sensitivity and allows subgrain boundaries >0.05–0.1° to be resolved depending on working distances, sample and grain sizes. Furthermore, the crystallography of the sample must be known in advance, which can pose a challenge for non-cubic systems.

Though the method optimizes only the completeness, complex grain shapes can be obtained as the grains assembling the microstructure are constrained by adjacent grains. Appropriate binarization of the diffraction contrast patterns is thus a critical and important step in order to avoid an over or under segmentation which jeopardizes completeness. The LabDCT reconstruction based on a simplified binary diffraction model reveals good agreement with and complements the well established absorption tomography reconstruction.

## 5. Conclusion   

A fast and versatile reconstruction scheme is presented, extending the recently introduced LabDCT imaging modality on a laboratory X-ray microscope to capture the crystallographic orientation and morphology of a polycrystalline microstructure. The shape of the grains obtained from LabDCT is verified for correctness by means of an absorption contrast reconstruction. Large grain maps can be stitched together from several reconstructions. Absorption and phase-contrast reconstructions, which are the base imaging modalities on the same X-ray microscope, can be combined with the LabDCT reconstruction, enabling correlative analysis. The LabDCT implementation closes the gap to synchrotron grain mapping and can routinely be used in the laboratory.
